# EEG Data Quality in Large-Scale Field Studies in India and Tanzania

**DOI:** 10.1523/ENEURO.0006-25.2025

**Published:** 2025-07-23

**Authors:** John-Mary Vianney, Shailender Swaminathan, Jennifer Jane Newson, Dhanya Parameshwaran, Narayan Puthanmadam Subramaniyam, Swaeta Singha Roy, Revocatus Machunda, Achiwa Sapuli, Santanu Pramanik, John Victor Arun Kumar, Pramod Tiwari, G. Nelson Mathews Mathuram, Laurent Boniface Bembeleza, Joyce Philemon Laiser, Winifrida Julius Luhwago, Theresia Pastory Maduka, John Olais Mollel, Neema Gadiely Mollel, Adella Aloys Mugizi, Isaac Lwaga Mwamakula, Raymond Edwin Rweyemamu, Upendo Firimini Samweli, James Isaac Simpito, Kelvin Ewald Shirima, Anand Anbalagan, Suresh Kumar Arumugam, Vinitha Dhanapal, Kanimozhi Gunasekaran, Neelu Kashyap, Dheeraj Kumar, Durgesh Pandey, Poonam Pandey, ArunKumar Panneerselvam, Sonam Rai, Porselvi Rajendran, Santhoshkumar Sekar, Oliazhagan Sivalingam, Prahalad Soni, Pushpkala Soni, Tara C. Thiagarajan

**Affiliations:** ^1^Centre for Human Brain and Mind (CEREBRAM), Nelson Mandela African Institute of Science and Technology (NMAIST), Arusha, Tanzania; ^2^Nelson Mandela African Institute of Science and Technology (NMAIST), Arusha, Tanzania; ^3^Sapien Labs Centre for Human Brain and Mind at Krea University, Chennai 600018, India; ^4^Institute for Financial Management and Research (IFMR), Chennai 600018, India; ^5^Sapien Labs, Arlington, Virginia 22209; ^6^Faculty of Medicine and Health Technology, Tampere University, Tampere 33520, Finland; ^7^LEAD at Krea University, Chennai 600113, India

**Keywords:** big data, data quality, EEG, high-throughput, India, Tanzania

## Abstract

There is a growing imperative to understand the neurophysiological impact of our rapidly changing and diverse technological, social, chemical, and physical environments. To untangle the multidimensional and interacting effects requires data at scale across diverse populations, taking measurement out of a controlled lab environment and into the field. Electroencephalography (EEG), which has correlates with various environmental factors as well as cognitive and mental health outcomes, has the advantage of both portability and cost-effectiveness for this purpose. However, with numerous field researchers spread across diverse locations, data quality issues and researcher idle time due to insufficient participants can quickly become unmanageable and expensive problems. In programs we have established in India and Tanzania, we demonstrate that with appropriate training, structured teams, and daily automated analysis and feedback on data quality, nonspecialists can reliably collect EEG data alongside various survey and assessments with consistently high throughput and quality. Over a 30 week period, research teams were able to maintain an average of 25.6 participants per week, collecting data from a diverse sample of 7,933 participants ranging from Hadzabe hunter-gatherers to office workers. Furthermore, data quality, computed on the first 5,831 records using two common methods, PREP and FASTER, was comparable to benchmark datasets from controlled lab conditions. Altogether this resulted in a cost per participant of under $50, a fraction of the cost typical of such data collection, opening up the possibility for large-scale programs particularly in low- and middle-income countries.

## Significance Statement

With wide human diversity, a rapidly changing environment, and growing rates of neurological and mental health disorders, there is an imperative for large-scale neuroimaging studies across diverse populations that can deliver high-quality data and be affordably sustained. Here we demonstrate, across two large-scale field data acquisition programs operating in India and Tanzania, that with appropriate systems, it is possible to generate high-throughput electroencephalography data of quality comparable with controlled lab settings. With effective costs of under $50 per participant, this opens new possibilities for low- and middle-income countries to implement large-scale programs and to do so at scales that previously could not be considered.

## Introduction

Understanding and parsing the multivariate and diverse environmental impacts on brain physiology requires large-scale, high-throughput studies that acquire data across diverse cross-sections of a population. One significant obstacle to this understanding is the ability to acquire high-quality electroencephalography (EEG) data at scale under diverse field conditions in a cost-efficient manner. Such data acquisition capacity is even more important today given the accelerated transformation of our technological, social, cultural, and physical environment (Anon, 2019; Arora, 2019; Roser et al., 2024). As an experience-dependent organ, the human brain is sensitive to change and variation in our stimulus environment. For example, EEG studies have demonstrated differences in resting-state and evoked potentials in response to interindividual differences in demographic profiles (Tomescu et al., 2018; Sandre et al., 2024), lifestyle habits (Khoo et al., 2024), developmental stages (Anderson and Perone, 2018; Wilkinson et al., 2024), and stimulus (Parameshwaran et al., 2019, 2021; Parameshwaran and Thiagarajan, 2023) or physical environments (Hou et al., 2023). How these changes impact our brain physiology is still poorly understood and have profound consequences for society.

While there are several larger-scale studies in progress, such as the Adolescent Brain Cognitive Development (ABCD) Study (Casey et al., 2018), Human Connectome Project (Van Essen et al., 2013), UK Biobank (Miller et al., 2016), Cuban Human Brain Mapping Project (Valdes-Sosa et al., 2021), Child Mind Institute’s Healthy Brain Network (Alexander et al., 2017), and ENIGMA Consortium (Thompson et al., 2020), they are typically resource intensive or require fixed infrastructure and consequently are able to acquire samples only on the scale of 10,000 or less, are geographically limited, and therefore do not reflect the breadth of human environment or culture. The ABCD project (Casey et al., 2018), for example, which utilizes fMRI as its primary neuroimaging device and captures data across 11,000 children each year in the United States has an annual budget of $41 million, which is prohibitive for most low- and middle-income countries. While affordable EEG devices are now available on the order of a few thousand dollars, a major aspect of cost is the need for trained or specialized technicians and research scientists, as well as ensuring sufficient throughput that minimizes idle time.

Here we present robust systems and processes for the cost-efficient, large-scale acquisition of high-quality EEG data across diverse field conditions by nonspecialist researchers that were developed and tested through pilot programs implemented at Sapien Labs’ Centers for Human Brain and Mind at Krea University in India and at the Nelson Mandela African Institute of Science and Technology (NM-AIST) in Tanzania. This approach addresses key challenges in field-based neuroscience research through three core components: (1) effective recruitment and training of nonspecialist field researchers; (2) real-time data quality monitoring using daily dashboards and feedback loops to quickly identify and address issues; and (3) streamlined participant recruitment and logistical coordination in the field. We describe the data throughput and resulting EEG data quality achieved in these programs from the first 3,413 and 2,418 participants across India and Tanzania, respectively. EEG data quality challenges primarily include eyeblink and movement artifacts and power line noise but can also include fluctuations in impedance and challenges with electrode placement due to varying hairstyles. We used two commonly available pipelines, preprocessing pipeline (PREP; Bigdely-Shamlo et al., 2015) and fully automated statistical thresholding (FASTER) (Nolan et al., 2010), to measure the percentage of bad channels and bad epochs and compared the results with EEG data from three highly cited benchmark datasets with equivalent experimental tasks obtained in a controlled lab environment with more expensive EEG devices (Singh et al., 2022; Wang et al., 2022; Miltiadous et al., 2023; Anjum et al., 2024; Xiang et al., 2024).

## Materials and Methods

### EEG equipment

**Figure 1.**
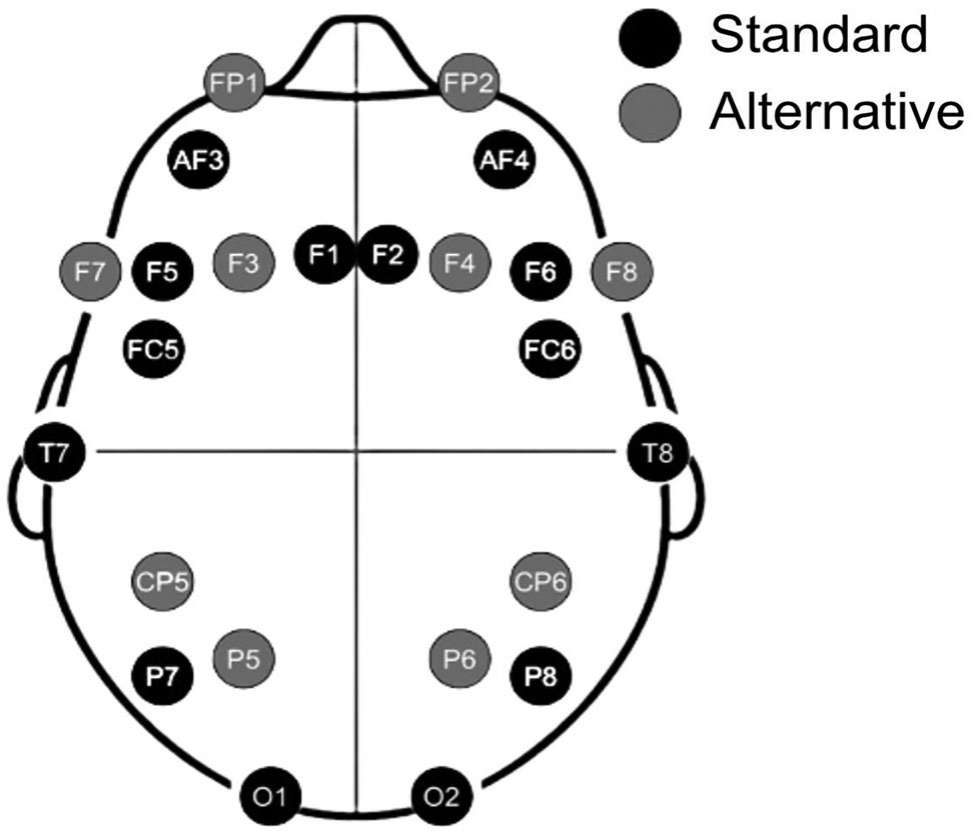
Electrode configuration used in the project. Black, standard configuration; gray, alternate nonstandard channels allowed in case of hair obstruction of a channel.

EEG was recorded with the wireless Emotiv FLEX 2 Gel headset using 16 out of 32 electrodes positioned according to the 10–20 international system and referenced to an ear clip sensor. The montage included eight electrodes over each hemisphere with alternative configurations utilized on occasion when the hair type or style imposed a restriction of data acquisition (e.g., braided buns; Fig. 1). The internal sampling rate was 2,048 samples per second downsampled to 256 Hz. Sixteen channels were selected as using 32 channels was found to more than double the setup time, making it a challenge to complete the full protocol and increasing the risk of participant dropout. Furthermore, we have found that 16 channels, and in some cases even 4 channels, are sufficient to predict various brain states and conditions (Parameshwaran et al., 2025; Subramaniyam and Thiagarajan, 2025).

### Research personnel

Field researchers were recruited among new college graduates as well as people with experience in field survey methods. All researchers were EEG naive and were trained in the use of the EEG device for 2 weeks prior to field data collection. A total of 12 field researchers were recruited in each country (India and Tanzania) and trained over 2 weeks. On Day 1, trainees were given a demonstration and explanation of EEG and performed hands-on trials with the help of a trainer who was experienced in EEG data acquisition. On Days 2 and 3, trainees performed hands-on resting–state EEG trials in pairs without direct assistance of the trainer and participated in problem-solving and debriefing sessions throughout the day. Each trainee collected EEG data from four to six people over the 2 d and received immediate feedback on data quality. Trainees then spent 1 week in the field, recording EEG from 10 to 15 participants under various circumstances (open-air rural locations, office rooms, etc.) and undertook debrief sessions at the end of the day to discuss challenges relating to field settings, equipment, and data quality and to identify solutions. Throughout the training, trainees were evaluated for their proficiency by the trainer. Trainees who were unable to reach minimum data quality and throughput numbers by the end of the training period were not hired. After the training, 93% met the standard and were retained. Trainees were compensated during the training period in line with university salary scales.

### Team structure

The 12 field researchers in each country were divided into teams of two or three. In addition, one participant recruitment manager (PRM) worked with field researchers in each region/country. The PRMs were recruited based on strong local networks as well as communication and organization skills and were responsible for identifying study participants and locations to fit with the sampling frame, reaching out to participants and ensuring that they were able to report to the study location at a specified time, as well as ensuring local government permissions and all logistics.

### Participant recruitment

Study participants were recruited in multiple locations in India and Tanzania according to a sampling frame designed to cover a broad range of income groups (low, medium, and high) across the lifespan, divided equally across biological males and females and divided among different types of geographies and settlements. In India, this covered multiple regions in the southern state of Tamil Nadu as well as the National Capital Region (Delhi, Haryana, Rajasthan, and Uttar Pradesh). In Tanzania, target locations spanned Arusha and Manyara regions including rural, suburban, and urban areas. Participants were age 18+ in Tanzania and age 13+ in India. Recordings were carried out in various locations including offices, schools, and open air. Participants were excluded only if they were unable to answer the questionnaire or carry out the tasks (see below).

All participants gave written informed consent and all procedures involving human subjects were approved by an ethical review board (India, IFMR Institutional Ethics Committee IFMR-IHEC/SL/0001/2023; National Ethics Committee Registry for Biomedical and Health Research, Department of Health Research; EC/NEW/INST/2023/3887; Tanzania, Kibong’oto Infectious Diseases Hospital–NM-AIST–Centre for Educational Development in health, Arusha–KNCHREC; KNCHREC00006/09/2023). A script developed to explain the study, the purpose of the study, and all other consent requirements accompanied the consent form. For those who could not read, the contents were read to them and any questions answered. For those who could not write, a thumbprint was obtained in lieu of a signature.

### Questionnaires and survey administration

An assessment of mind health and well-being, the MHQ (Newson and Thiagarajan, 2020; Newson et al., 2022), and extensive demographic, life context, and lifestyle questions were administered along with the EEG protocol. This included lifestyle aspects such as sleep, exercise and diet, family relationships, technology use, substance use, traumatic experiences, and medical conditions. Those with familiarity and ease with reading and digital devices were given a tablet from which they could complete the questions on their own. For those who were low-literate or had difficulty with manipulating an electronic device, the questions were administered by the researchers. Depending on the mode of collection, the questionnaires took anywhere from 30 min to 1 h to complete. A session form taking ∼3 min to complete was also administered prior to the start of EEG recording (see EEG protocol below).

### Field EEG protocol and data quality monitoring

Resting-state EEG was collected when participants were sitting quietly with their eyes closed (EC) for 3 min and eyes open (EO) for 3 min. During the EO task, participants were instructed to look at their surroundings, rather than the laptop or the researcher. In addition, all participants completed a Raven’s progressive matrix task (TASK) (Raven, 2000). A session information questionnaire was administered prior to the start of recording and queried the mental and physical status of the participant including physical symptoms (e.g., headache, cold, stomach ache), any medications they had taken in the past 24 h, any substances such as caffeine or drugs consumed in the past 12 h, time of the last meal, duration of previous night’s sleep, and time since they woke up as well as their mood and alertness at the time of recording.

Data quality was monitored in real-time by researchers with end-of-day reports returned to each field researcher. In addition, with channel quality metrics available in Emotiv’s recording software, scripts were run on test data obtained after electrode positioning to compute data quality and indicate any adjustments needed. Experimental protocols were initiated only after the test signal passed this quality test. In addition, postrecording, all data were automatically analyzed for signal quality using the FASTER *Z*-score criteria. A dashboard showing both throughput and signal quality could be viewed by the field researchers and supervising staff for immediate course correction in case of arising data quality issues (Table 1). For each research team, a breakup by the EEG device number was also provided as a next level of detail to determine if any data quality issues arose due to the device itself.

**Table 1.** Daily report provided to field research team and supervisors in India

Report date										
All teams	# recordings		# nonstandard channels		# missing channels (out of 16)		% bad channels		% bad epochs	
Task	On date	YTD	On date	YTD	On date	YTD	On date	YTD	On date	YTD
Overall	30	230	0	1	1	2	4.5%	4.2%	10%	10%
Eyes Closed	30	230	0	1	1	2	3.3%	2.6%	8.9%	8.7%
Eyes open	29	230	0	1	1	2	3.4%	4.3%	10.8%	10.4%
Ravens's Task	29	226	0	1	1	2	6.9%	5.8%	11.5%	11.2%
Eyes closed	# recordings		# nonstandard channels		# missing channels (out of 16)		% bad channels		% bad epochs	
Research team	On date	YTD	On date	YTD	On date	YTD	On date	YTD	On date	YTD
NCR1	10	77	0	1	2	1	0.0%	2.6%	8.9%	9.9%
NCR2	8	63	0	0	1	2	12.5%	4.8%	8.1%	8.9%
NCR3	12	90	0	0	0	2	0.0%	1.1%	9.0%	8.1%
TN1	3	310	0	1	2	3	3.3%	4.5%	2.9%	2.9%
TN2	5	493	1	2	0	1	3.4%	6.4%	2.6%	2.4%
TN3	5	595	0	0	0	0	6.9%	3.2%	5.7%	3.5%

TN, Tamil Nadu Team; NRC, National Capital Region; YTD, year to date.

### Benchmark EEG datasets

To facilitate the comparison of the quality of our EEG recordings with EEG datasets acquired in a more controlled setting, we compared the results of EEG quality metrics for each condition (EO, EC, or TASK) against benchmark datasets obtained from OpenNeuro or NEMAR which we refer to as BM1, BM2, and BM3 (Singh et al., 2022; Wang et al., 2022; Miltiadous et al., 2023; Anjum et al., 2024; Xiang et al., 2024). These represent highly cited datasets that are openly available and described in Table 2.

**Table 2.** Details on conditions and tasks used compared with benchmark datasets BM1, BM2, and BM3 used in this study to compare EEG data quality

Condition	Study protocol	BM1	BM2	BM3
Eyes open	Three minutes of 16-channel EEG with eyes open and looking ahead	Three minutes of 64-channel EEG from 49 participants (Anjum et al., 2024)	Five minutes of 64-channel EEG data from 60 participants (Wang et al., 2022)	Five minutes of 61-channel EEG data from 71 participants (Xiang et al., 2024)
Eyes closed	Three minutes of 16-channel EEG with eyes closed	19-channel EEG recordings from 29 participants (Miltiadous et al., 2023)	Five minutes of 64-channel EEG data from 60 participants (Wang et al., 2022)	Five minutes of 61-channel EEG data from 71 participants (Xiang et al., 2024)
Task	Two to five minutes of 16-channel EEG when performing a Raven's progressive matrix task	Sixty-four–channel EEG data from 60 participants performing mathematics task (subtraction; Wang et al., 2022)	Sixty-four–channel EEG data from 60 participants performing mathematics task (memory task involving recollecting events of the day; Wang et al., 2022)	Sixty-nine–channel EEG from 23 participants performing working memory task involving memorizing and ignoring a set of presented letters (modified Sternberg task; Singh et al., 2022)

### EEG data quality analysis

The quality of EEG recordings from India (EC, *N* = 3,402; EO, *N* = 3,413; TASK, *N* = 3,241) and Tanzania (EC, *N* = 2,418; EO, *N* = 2,410; TASK, *N* = 2,381) was evaluated using two commonly accepted approaches: (1) FASTER (Nolan et al., 2010) and (2) PREP (Bigdely-Shamlo et al., 2015). Each EEG recording was evaluated for the percentage of bad epochs and bad channels based on the criteria proposed by the FASTER and PREP methods. For detecting bad epochs, the EEG data were divided into epochs of 2 s. The EEG recordings were high-pass filtered at 0.5 Hz before the detection of bad channels and epochs. As data acquisition is ongoing, the reported *N* values for each analysis reflect the total number of records acquired up to the day the analysis was initiated.

#### Detection of bad channels by FASTER

Detection of bad channels was based on three parameters (Nolan et al., 2010) which included the following:

1. A mean correlation coefficient between channels pairs with *Z*-score >3 implying non-EEG signal contamination
2. A signal variance *Z*-score >3
3. Hurst exponent with *Z*-score >3

In addition to these three criteria, we also assessed contamination with powerline noise. To this end, channels with a mean power *Z*-score >3 between 48 and 62 Hz were flagged as bad channels.

#### Detection of bad channels by PREP

Criteria used in the PREP method to identify bad or unusable channels were based on the following:

1. EEG channels with flat signals (threshold <1 × 10^−15^ μV) and NaN values or channels with a flat signal (<1 × 10^−15^ μV) for >1% of windows
2. Amplitudes that exceeded a robust *Z*-score >5 (as compared with standard *Z*-score by the FASTER method; Nolan et al., 2010)
3. A correlation threshold of <0.4 between >1% of all 2 s windows in the signal
4. The ratio of high- (>50 Hz) and low-frequency components exceeding a robust *Z*-score of 5 where a 50 Hz low-pass finite impulse response filter was used to separate the low- and high-frequency components

#### Detection of bad epochs by FASTER

Criteria used by the FASTER method were based on the following:

1. An amplitude range transformed *Z*-score >3, where the amplitude range was calculated as the difference between the maximum and minimum value in each epoch.
2. Variance within an epoch having a *Z*-score >3 (used in order to detect artifacts due to participant movement).
3. *Z*-score of the deviation parameter for an epoch >3, where deviation parameter measured the deviation of an epoch's average value (across time) from the average values across all channels. For *N* epochs, this resulted in *N* × *M* deviation values, where *M* was the number of EEG channels. The deviation parameter values were then averaged across *M* channels resulting in *N* deviation parameters.

#### Detection of bad epochs by PREP

We modified the PREP method (Bigdely-Shamlo et al., 2015) to also detect bad epochs. For each EEG channel and epoch, robust standard deviation was computed using the interquartile range and multiplying it by 0.7413. For each channel and epoch, the median values were also computed, following which a robust *Z*-score was obtained for each epoch in each channel. A maximum robust *Z*-score >5 for each epoch across all the channels was marked as a bad epoch.

### Code accessibility

The FASTER method was implemented in-house in Python, following the approach described by Nolan et al. (2010). In the case of PREP, the Python code provided by the PREP developers was adapted for our PREP. The code was implemented on a MacBook Air 15 M4 using a macOS Sequoia operating system. The code/software described in the paper is freely available online at https://github.com/narayanps/SapienLabsDataQuality. The code is available as Extended Data.

### Comparison of conditions

Within our data, we made comparisons between multiple recording conditions including recordings conducted indoor versus outdoor and during summer versus winter months. In India, summer months considered were May and June and winter months December to February, while in Tanzania, summer (or warmer) months were November to February and winter months were June to October. In addition, while the hair type was not specifically recorded, differences between males and females were also compared where females would, on average, have longer hair.

### Statistical analysis

In addition to mean and standard deviation values, we computed statistical significance of differences as follows: For comparisons of the percentage of bad channels and bad epochs between our data and benchmark data, given the large size of our data compared with benchmark datasets, we used a bootstrap approach comparing the benchmark data to randomly selected samples of the same size from our datasets. Reported statistical significance is the average *p* value across 50 such iterations. For comparisons of the percentage of bad channels across conditions within our data, we report *p* values using a standard *t* test.

### Calculation of peak alpha frequency

Peak alpha frequency was computed for the resting EC condition by identifying the peak value within the alpha range (7–12 Hz) in the power spectral density, computed using the Pwelch function with a 2 s window and 50% overlap.

## Results

**Figure 2.**
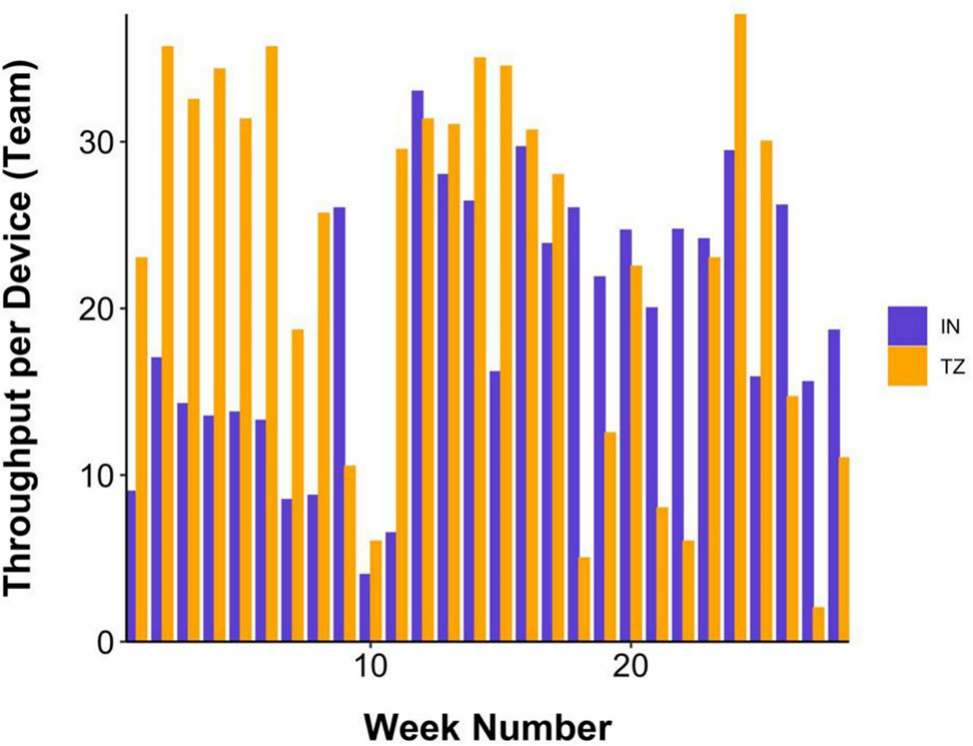
Weekly throughput for the India (IN) and Tanzania (TZ) pilot phase calculated as participants recorded per EEG device or research team.

Figure 2 shows the weekly throughput per EEG device for the first 30 weeks of data collection for the India and Tanzania teams. Weekly throughput was calculated as the number of participants recorded per EEG device per week since the first week post-training, where each device was managed by a team of 2–3 field researchers (average 2.25). Only participants for whom the full EEG and survey protocol was completed were included (total of 1 h per participant). On average, 25.6 participants were recorded per device per week.

### Percentage of bad channels

**Figure 3.**
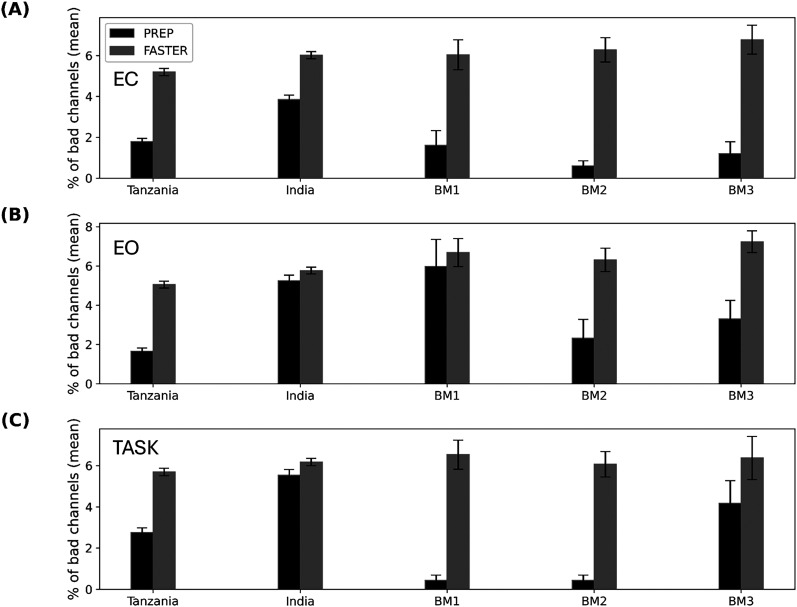
The average percentage of bad channels for Tanzania, India, and benchmark (BM1, BM2, and BM3) EEG recordings for (***A***) EC, (***B***) EO, and (***C***) TASK conditions using PREP (black) and FASTER (gray) method. Error bars indicate standard error of the mean. CDFs are shown in Extended Data Figure 3-1.

Figure 3 shows the mean percentage of bad channels based on PREP and FASTER for all data from Tanzania, India, and each of the three benchmark datasets [BM1–BM3 (Singh et al., 2022; Wang et al., 2022; Miltiadous et al., 2023; Anjum et al., 2024; Xiang et al., 2024); cumulative distribution functions, CDFs, shown in Extended Data Fig. 3-1]. Specifically, in the EC condition, the percentage of bad channels (Fig. 3*A*) was slightly higher in the field samples compared with benchmarks using the PREP method. Differences that were statistically significant included Tanzania versus BM2 (Tanzania, 1.87 ± 0.41%; BM2, 0.6 ± 0.13%; *p* < 0.05) and India versus BM1 (India, 3.93 ± 1.01%; BM1, 1.61 ± 0.36%; *p* < 0.02) and BM2 (India, 3.93 ± 1.01%; BM2, 0.06 ± 0.36%; *p* < 0.01) for PREP method, with benchmark data having a lower percentage of mean bad channels compared with field data. On the other hand, the percentage of bad channels was higher but comparable between both field samples and benchmarks using FASTER (Tanzania, 5.19 ± 0.0.09%; India, 6.02 ± 0.08%; BM1, 6.04 ± 0.37%; BM2, 6.28 ± 0.3%; BM3, 6.77 ± 0.35%).

10.1523/ENEURO.0006-25.2025.f3-1 Figure 3-1 Cumulative distribution for the percentage of bad channels for FASTER (left) and PREP (right) for India, Tanzania and benchmark (BM) EEG datasets. Each row represents the EEG condition which include Eyes closed (EC, top), Eyes open (EO, middle) and TASK (bottom). Download Figure 3-1, TIF file.

For the EO condition (Fig. 3*B*), the percentage of bad channels was higher overall compared with EC for almost all datasets but lower in the field data compared with benchmarks as follows: Tanzania data were similar to BM2 (Tanzania, 1.68 ± 0.40%; BM2, 2.32 ± 0.95%) but significantly lower than BM1 (5.96 ± 1.38%; *p* < 0.01) and lower than BM3 (3.30 ± 0.94%), while the India data (5.33 ± 0.46%) were comparable with BM1. Using the FASTER method, the Tanzania data had a significantly lower (*p* < 0.01) percentage of bad channels (5.10 ± 0.52%) compared with all the benchmark datasets (between 6 and 8%), while the percentage of bad channels between the India data and the benchmarks was comparable.

In the case of the TASK condition (Fig. 3*C*), BM1 and BM2 had a significantly lower percentage of bad channels (*p* < 0.05) using the PREP method (0.44 ± 0.13% and 0.43 ± 0.24%, respectively) compared with Tanzania (2.78 ± 1.01%) and India datasets (5.17 ± 0.63%), while BM3 was similar (4.16 ± 1.1%). Using the FASTER method, we obtained comparable values across all datasets with the percentage of bad channels ranging between 5.5 and 6.5%.

### Percentage of bad epochs

**Figure 4.**
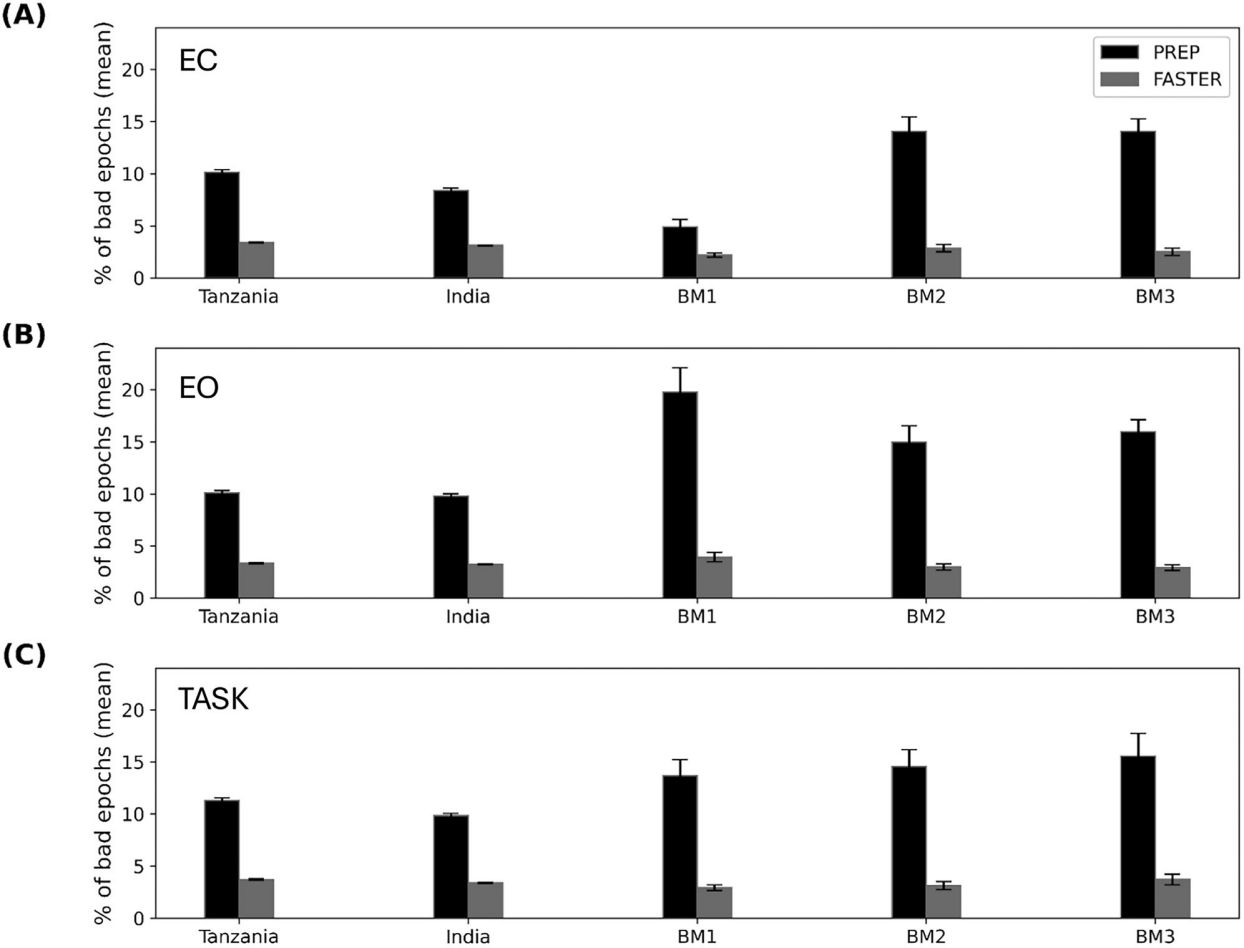
The average percentage of bad epochs for Tanzania, India, and benchmarks (BM1, BM2, and BM3). EEG recordings for (***A***), EC, (***B***) EO, and (***C***) TASK conditions using PREP (black) and FASTER (gray) method. The error bars indicate the standard error of the mean. CDFs are shown in Extended Data Figure 4-1.

Figure 4 shows the average percentage of bad epochs based on PREP and FASTER for all data from Tanzania, India, and each of the three benchmark datasets (BM1–BM3; CDFs shown in Extended Data Fig. 4-1). Here the field data had a lower percentage of bad epochs across almost all conditions using PREP and a comparable percentage of bad epochs, compared with the benchmarks using FASTER.

10.1523/ENEURO.0006-25.2025.f4-1 Figure 4-1 Cumulative distribution for the percentage of bad epochs for FASTER (left) and PREP (right) for India, Tanzania and benchmark (BM) EEG datasets. Each row represents the EEG condition which include Eyes closed (EC, top), Eyes open (EO, middle) and TASK (bottom). Download Figure 4-1, TIF file.

For the EC condition (Fig. 4*A*), the percentage of bad epochs for Tanzania and India data was significantly lower than BM2 and BM3 (*p* < 0.05) using PREP, similar to using FASTER, but higher compared with BM1 using both PREP and FASTER, with the percentage of bad epochs ranging between 2 and 3% (PREP, Tanzania, 10.31 ± 1.05%; India, 8.41 ± 0.1.05%; BM2, 14.07 ± 0.71%; BM3, 14.05 ± 0.62%; BM1, 4.91 ± 0.37%; FASTER, India, 3.10 ± 0.22%; Tanzania, 3.39 ± 0.25%; BM1, 2.20 ± 0.11%).

For EO and TASK conditions (Fig. 4*B*,*C*, respectively), the India and Tanzania datasets had a considerably lower percentage of bad epochs compared with benchmarks with the PREP method and generally comparable results with FASTER. In the case of EO, differences that were statistically significant (*p* < 0.05) included, for PREP method, Tanzania (10.22 ± 0.68%) and India (9.92 ± 0.77%) versus BM1 (19.76 ± 1.19%), BM2 (14.96 ± 0.81%), and BM3 (15.96 ± 0.60%), where both Tanzania and India recordings had a significantly lower percentage of bad epochs compared with the benchmark data. For TASK, in the case of PREP, India (10.10 ± 1.34%) and Tanzania (11.49 ± 1.30%) had a significantly lower percentage of bad epochs compared with BM2 (14.57 ± 0.82%) and BM3 (15.56 ± 0.60%), while for the FASTER method, only Tanzania (3.76 ± 0.39%) had a significant higher percentage of bad epochs compared with BM1 (2.93 ± 0.14%).

### Difference between conditions

We next compared the percentage of bad channels during the EC condition between measurements conducted in females, who on average tend to have longer hair, versus males (Table 3). There was no significant difference between males and females in any location.

**Table 3.** The percentage of bad channels using PREP and FASTER during EC condition between recordings from males and females

Method	Country	Male	Female	*p* value
PREP	Tanzania	1.86 ± 0.11	1.78 ± 0.1	0.61
PREP	India	5.6 ± 0.67	6.71 ± 0.63	0.23
FASTER	Tanzania	5.41 ± 0.13	5.09 ± 0.12	0.06
FASTER	India	7.21 ± 0.37	7.14 ± 0.33	0.85

We similarly compared the percentage of bad channels in recordings conducted at indoor and outdoor locations during summer versus winter months (Table 4). We note that temperature differences across the year are not substantial in either Tanzania (average of 80–85°F in summer months and 70–75°F during winter months in the Arusha region) or Tamil Nadu in South India (90–100°F in summer and 80–84°F in winter), while Delhi has a greater range (averages of 90–100°F in summer months and 60–70°F in winter). No recordings were conducted outdoors in the winter months in the Delhi region. The overall percentage of bad channels was significantly higher for indoor recordings during the summer months in Tanzania using both PREP and FASTER, during the winter months in the Delhi region using PREP, and during the summer months using FASTER. This indicated no consistent pattern, suggesting that the differences may not pertain to the weather per se.

**Table 4.** The percentage of bad channels using PREP and FASTER during the EC condition between recordings conducted at indoor versus outdoor locations during summer and winter months

Method	Location	Country	Summer	Winter	*p* value
PREP	Indoor	Tanzania	2.03 ± 0.21	1.22 ± 0.13	<0.001
PREP	Indoor	India-TN region	2.95 ± 0.20	2.10 ± 0.47	NS
PREP	Indoor	India-Delhi region	6.74 ± 0.65	12.03 ± 1.19	<0.001
PREP	Outdoor	Tanzania	1.74 ± 0.16	1.96 ± 0.12	NS
PREP	Outdoor	India-TN region	2.53 ± 0.85	3.57 ± 2.06	NS
PREP	Outdoor	India-Delhi region	4.76 ± 2.38	NA	NA
FASTER	Indoor	Tanzania	5.54 ± 0.22	4.84 ± 0.18	<0.05
FASTER	Indoor	India-TN region	5.91 ± 0.21	5.73 ± 0.48	NS
FASTER	Indoor	India-Delhi region	8.29 ± 0.37	5.99 ± 0.42	<0.001
FASTER	Outdoor	Tanzania	5.01 ± 0.18	5.46 ± 0.14	NS
FASTER	Outdoor	India-TN region	5.07 ± 0.95	8.93 ± 1.79	NS
FASTER	Outdoor	India-Delhi region	4.76 ± 2.38	NA	NA

NS, not significant; TN, Tamil Nadu.

### Comparison of peak alpha frequency

**Figure 5.**
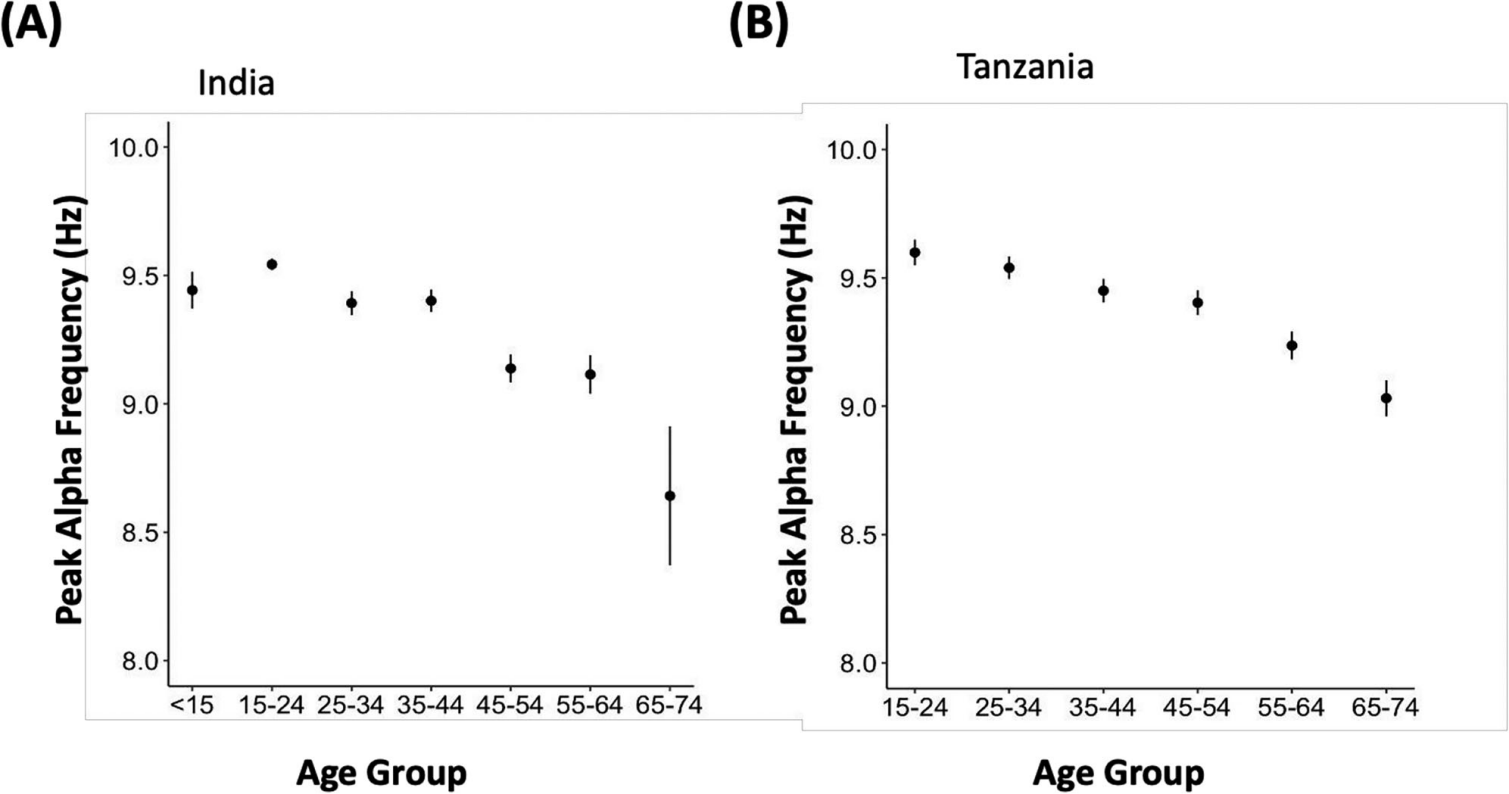
Peak alpha frequency (mean ± SEM) by age for (***A***) India (*N* = 3,402) and (***B***) Tanzania (*N* = 2,418).

Finally, we looked at a key feature of the EEG—the frequency of the alpha oscillation. This was identified as the frequency associated with the peak in the power spectrum in the alpha band in the EC resting condition. This feature of the EEG has been previously shown to increase with age, up to age 15, and then decrease with age after the age of 25 or 30 (Chiang et al., 2011; Joffe et al., 2021). Consistent with this trend, we show that the peak alpha frequency declined with age from a mean of 9.5 ± 0.02 Hz to 8.6 ± 0.23 Hz from the age group 15–24 to 65–74 in India and from 9.6 ± 0.05 Hz to 9.0 ± 0.07 Hz in Tanzania (Fig. 5). In India, where data were available for age 13–15, the peak alpha frequency was lower than age 15–24, at 9.4 ± 0.07 Hz.

## Discussion

Here we have shown that, with robust systems and processes, it is possible to affordably collect high-throughput, high-quality EEG data across diverse field locations with training of an EEG naive field research team. This opens up a new frontier for research of the impacts of rapid environmental change on diverse populations in diverse environments that is so crucially needed (Henrich et al., 2010; Dotson and Duarte, 2020). It also overcomes the practical and cost constraints associated with other types of neuroimaging infrastructure in low- and middle-income countries (Geethanath and Vaughan, 2019; Arnold et al., 2023).

### Data quality parameters

The percentage of bad channels in the field data was slightly higher than the benchmark data using PREP but comparable using FASTER. The percentage of bad channels was also higher in FASTER overall compared with PREP. This is because FASTER uses a standard amplitude *Z*-score of 3 as the threshold for detection, compared with a robust *Z*-score threshold of 5 for PREP. This means that, while the field data had a comparable number of channels that met the >3 threshold, it had a larger number of channels that met the >5 threshold of the robust *Z*-score. We also note that the field EEG uses just 16 channels, while the benchmark datasets use 64 channels. Thus, a single bad channel is equivalent to 6.25% in the field data but only 1.6% in the benchmark data.

In contrast, the percentage of bad epochs using PREP was comparable between the field data and two of the benchmark datasets but lower than one of them (BM1). The PREP method is traditionally used for bad channel detection and has been adapted to detect bad epochs, where if a robust *Z*-score of 5 for an epoch was exceeded in even one channel, it is marked as a bad epoch. In FASTER, the percentage of bad epochs was lower compared with PREP and comparable across the field and benchmark data. This is because it uses the average across all channels as the threshold for metrics such as the amplitude range and variance. While channels may be eliminated due to artifacts, channels with bad epochs can still provide useful information. In fact, recent studies suggest that artifact removal can actually worsen results as it likely removes substantial regions of useful signal along with the artifacts (Delorme, 2023).

### Data quality considerations

It is of substantial reassurance that there were generally no significant data quality differences between EEG recordings obtained in indoor versus outdoor environments and that overall, there was no consistent pattern of differences in data quality between seasons. However, on some days with very high temperatures (over 100°F) during the summer months in India, the gel tends to melt, and excess sweat may also impair the signal. Thus, recordings were typically moved indoors on these days. Increased temperatures due to climate change may thus impose on substantial cost on such data acquisition.

In addition to field conditions and researcher practices, another key quality consideration in large-scale, long-term data collection is the deterioration of various components of the EEG equipment. This includes stretching of the holes on the caps leading to movement of electrodes, as well as residue buildup on the electrodes. In addition to regular inspection of devices and peripheral equipment, we also track data quality by device and peripherals which are numbered and labeled. This helps identify when declining data quality is due to device deterioration rather than researcher error, allowing faulty parts to be replaced promptly. Generally, cap replacement is necessary after every 50–100 recording sessions, while electrode replacement is necessary after every 500–1,000 recording sessions.

Finally, we also note several other data quality factors, beyond the EEG signal, that have to be considered, such as correct capturing of channel names and other meta-data that is important for interpretation of the signal. While this is not shown here, these elements are also critically important aspects of the daily monitoring and feedback required to generate high-quality data at scale.

### From lab to scale

Going from small to large scale without compromising data quality and doing so at reasonable cost is a challenge in many domains. While small lab studies are typically supervised by a PI along with students and post docs focused on the quality of their own study, large-scale studies require a different paradigm. With a large number of people involved, the considerations for scale include standardization of methodologies, effective training methods, and team structures as well as dashboards with daily analysis and feedback for rapid trouble shooting. The quality of data is thus more a reflection of the effectiveness of these processes over other factors such as researcher skill and device quality. In the absence of such processes, it is possible that issues may not be detected until much later, with data for many participants having to be discarded. This will result in large costs due to wastage. With the throughput rates and data quality accomplished here, costs can be as low as $50/participant for a 1 h protocol that includes survey and EEG. Based on this experience, standard operating and training manuals are being developed to enable a standardized training for new recruits within existing teams and expansions into new geographies.

### The Sapien Center datasets and potential applications

The large-scale data acquired here represent the pilot phase of an ongoing study that explores how changing human environments and the diversity of human experience differentially impact brain physiology and functioning. In the first 6 months of this pilot, teams of 12 field researchers in two countries have already generated the largest database of general population EEG recordings in their countries or continents (*N* = 5,500 and *N* = 6,200 for Tanzania and India, respectively). The datasets include not just the EEG recordings described here but also extensive assessment of mental well-being or mind health along with a vast array of lifestyle, life experience, and environmental factors. Given the scale, these data will allow for various analysis of the relationship between environmental factors and brain physiology and how they differ across populations.

In this study, we show the change in peak alpha frequency by age as an example of both data quality and the potential of the dataset. The results are consistent with the literature where peak alpha frequency has been shown to increase across childhood and decrease over adulthood (Chiang et al., 2011; Joffe et al., 2021). However, we note that the pattern and rate of decrease differs between Tanzania and India and is shifted relative to trends in Western datasets, already pointing to possible population differences that may be mediated by environmental factors.

## Data Availability

We anticipate that this data and associated training materials will become dynamically available to the research community by the end of 2025 through our data platform Brainbase. This will include raw data as well as numerous standard and novel metrics computed from the EEG, along with survey elements. In the meantime, data are available on request. Sample EEG data from a subset of participants are freely available online (https://github.com/narayanps/SapienLabsDataQuality) and are available as Extended Data 2.

10.1523/ENEURO.0006-25.2025.d1 Data 1 Python code for the implementation of the FASTER and PREP methods used in this study. Download Data 1, ZIP file.

10.1523/ENEURO.0006-25.2025.d2 Data 2 Sample EEG data from a small subset of participants from Tanzania and India across the 3 conditions (EO, EC, TASK). Download 2, ZIP file.

## Synthesis

Reviewing Editor: Ifat Levy, Yale School of Medicine Decisions are customarily a result of the Reviewing Editor and the peer reviewers coming together and discussing their recommendations until a consensus is reached. When revisions are invited, a fact-based synthesis statement explaining their decision and outlining what is needed to prepare a revision will be listed below. The following reviewer(s) agreed to reveal their identity: Faisal Mushtaq, Guiomar Niso. Note: If this manuscript was transferred from JNeurosci and a decision was made to accept the manuscript without peer review, a brief statement to this effect will instead be what is listed below.

In this paper, the authors show that large-scale EEG data collection in diverse field settings is feasible with proper training, structured teams, and automated data quality monitoring. They demonstrate this through programs in India and Tanzania, where non-specialists successfully collected high-quality EEG data from 7,933 participants over 30 weeks, maintaining an average of 25.6 subjects per week. The data quality, assessed using PREP and FASTER, was comparable to benchmark datasets from controlled lab settings, while the cost per subject was significantly reduced.

This is an impressive project and a major logistical feat! Although the data are not currently shared openly, the study provides valuable insights into data quality metrics and methodologies. This study highlights the potential for scalable EEG research in low- and middle-income countries. Overall, this is a valuable work and the writing is clear. However, there are several points that are either missing or require some further clarification:

- The goal of the work is not very focused on the initial paragraphs. The introduction will be stronger if it focuses just on the most relevant ideas and the main contribution the paper is making.

- The validation claims are based on two automated pipelines and artifact rejection measures. Providing additional analysis of the actual underlying signal (e.g. alpha suppression effect size) would offer a more comprehensive assessment of data quality beyond bad segments and channels. This could be benchmarked against the rsEEG datasets already preprocessed.

- Why use a task dataset as a benchmark when you are only reporting resting state? Although eyes open is sometimes referred to as a task, it is more commonly treated as a resting state.

- "During the EO task, participants were instructed to look at their surroundings, rather than the laptop or the researcher." - what was the reason for these instructions?

- Field EEG studies introduce challenges, such as movement artifacts, power line noise, and variations in hair type affecting electrode placement. While the authors implement real-time monitoring, they should clarify how these issues were consistently addressed across different teams and settings. Importantly, including an analysis of environmental factors that impact data quality would enhance the manuscript's contribution.

- Add subtitles in figures 3 and 4, for each of the panels A, B and C. Why were there no statistical tests? Could these results benefit from some statistics to compare groups and conditions?

- Please indicate how you accessed and computed FASTER and PREP algorithms? Through EEGLAB plugins / Through code available somewhere?

These methods are over a decade old. Have you explored more recent tools that incorporate advancements or offer improved performance? Given the study's focus on data quality, addressing this aspect in more depth could enhance the work.

- Have you attempted to compare the results of FASTER and PREP with a professional's visual inspection for a small sample? As the authors may be aware, automated methods are not always fully reliable or optimal for EEG data and artifact rejection.

- Have you looked at other data quality metrics, for example those based on spectral content or others?

- The discussion section could elaborate more on what the authors have learnt from this unique experience. For example, describing challenges of long term data collection, potential sources of signal drift, and strategies being implemented for addressing them would strengthen the manuscript.

Minor comments:

- Why use only 16 channels when you are applying a 32 channel system? Is it because of limited time? If so, this should be explicitly stated.

- The authors report that they have collected 7,933 recordings in total, but their validation focused on less than half of that number. Given the automated nature of the analysis, it seems like a larger pool of the data could have been analyzed. Could you explain why and how this subset was selected? Simply the first batch of recordings? Or randomly selected?

- Is training standardized using general guidelines or common materials?

- Lines 138-140: "trainees were evaluated for their proficiency by the trainer. Trainees who were unable to reach minimum data quality and throughput numbers by the end of the training period were not hired." How did you evaluate the trainees? Do you have a general criteria common to all? Did they receive any monetary compensation for the work? If so, approximately how much?

- Clearer explanations of participant inclusion and exclusion criteria would be useful.

- Ethical approvals are briefly mentioned, but more detail on informed consent procedures, especially given the demographics, is necessary. How did you ensure *informed* consent was provided?

- How were questionnaire data collected? Online/verbally/paper? How long does it take?

- While the dataset seems to be planned for release in 2025, clearer guidelines on metadata documentation would be beneficial. A large-scale project like this would benefit from data standardization to enhance FAIR principles (Findability, Accessibility, Interoperability, and Reusability). However, there is no mention of established neuroimaging standards, such as the Brain Imaging Data Structure (BIDS), which could improve data organization and sharing. Also please note there are two statements on data availability.

## References

[B1] Alexander LM, et al. (2017) An open resource for transdiagnostic research in pediatric mental health and learning disorders. Sci Data 4:170181. 10.1038/sdata.2017.181 29257126 PMC5735921

[B2] Anderson AJ, Perone S (2018) Developmental change in the resting state electroencephalogram: insights into cognition and the brain. Brain Cogn 126:40–52. 10.1016/j.bandc.2018.08.001 30144749

[B3] Anjum MF, Espinoza AI, Cole RC, Singh A, May P, Uc EY, Dasgupta S, Narayanan NS (2024) Resting-state EEG measures cognitive impairment in Parkinson’s disease. NPJ Parkinsons Dis 10:1–13. 10.1038/s41531-023-00602-0 38172519 PMC10764756

[B4] Anon (2019) Davos 1973 to Davos 2020: how the world economy has changed. World Economic Forum. Available at: https://www.weforum.org/agenda/2019/12/how-has-global-economy-changed-50-years-davos-1973-to-2020-world-economic-forum/. Accessed September 17, 2024.

[B5] Arnold TC, Freeman CW, Litt B, Stein JM (2023) Low-field MRI: clinical promise and challenges. J Magn Reson Imaging 57:25–44. 10.1002/jmri.28408 36120962 PMC9771987

[B6] Arora NK (2019) Earth: 50 years challenge. Environ Sustain 2:1–3. 10.1007/s42398-019-00053-5

[B7] Bigdely-Shamlo N, Mullen T, Kothe C, Su K-M, Robbins KA (2015) The PREP pipeline: standardized preprocessing for large-scale EEG analysis. Front Neuroinform 9:16. 10.3389/fninf.2015.00016 26150785 PMC4471356

[B8] Casey BJ, et al. (2018) The adolescent brain cognitive development (ABCD) study: imaging acquisition across 21 sites. Dev Cogn Neurosci 32:43–54. 10.1016/j.dcn.2018.03.001 29567376 PMC5999559

[B9] Chiang AKI, Rennie CJ, Robinson PA, van Albada SJ, Kerr CC (2011) Age trends and sex differences of alpha rhythms including split alpha peaks. Clin Neurophysiol 122:1505–1517. 10.1016/j.clinph.2011.01.040 21349761

[B10] Delorme A (2023) EEG is better left alone. Sci Rep 13:2372. 10.1038/s41598-023-27528-0 36759667 PMC9911389

[B11] Dotson VM, Duarte A (2020) The importance of diversity in cognitive neuroscience. Ann N Y Acad Sci 1464:181–191. 10.1111/nyas.14268 31663150

[B12] Geethanath S, Vaughan JT Jr (2019) Accessible magnetic resonance imaging: a review. J Magn Reson Imaging 49:e65–e77. 10.1002/jmri.26638 30637891

[B13] Henrich J, Heine SJ, Norenzayan A (2010) Most people are not WEIRD. Nature 466:29. 10.1038/466029a 20595995

[B14] Hou J, Wang C, Jia L, Ma H (2023) Long-term exposure to high altitude reduces alpha and beta bands event-related desynchronization in a Go/NoGo task. Sci Rep 13:19719. 10.1038/s41598-023-45807-8 37957177 PMC10643632

[B15] Joffe D, Oakley DS, Lucini FA, Palermo FX (2021) Measurements of EEG alpha peak frequencies over the lifespan: validating target ranges on an in-clinic platform. 2021.10.06.463353. Available at: https://www.biorxiv.org/content/10.1101/2021.10.06.463353v2. Accessed April 28, 2025.

[B16] Khoo SY, Lai WH, On SH, On YY, Adam BM, Law WC, Ng BHS, Fong AYY, Anselm ST (2024) Resting-state electroencephalography (EEG) microstates of healthy individuals following mild sleep deprivation. Sci Rep 14:16820. 10.1038/s41598-024-67902-0 39039219 PMC11263689

[B17] Miller KL, et al. (2016) Multimodal population brain imaging in the UK Biobank prospective epidemiological study. Nat Neurosci 19:1523–1536. 10.1038/nn.4393 27643430 PMC5086094

[B18] Miltiadous A, et al. (2023) A dataset of scalp EEG recordings of Alzheimer’s disease, frontotemporal dementia and healthy subjects from routine EEG. Data 8:95. 10.3390/data8060095

[B19] Newson JJ, Pastukh V, Thiagarajan TC (2022) Assessment of population well-being with the mental health quotient: validation study. JMIR Ment Health 9:e34105. 10.2196/34105 35442210 PMC9069309

[B20] Newson JJ, Thiagarajan TC (2020) Assessment of population well-being with the mental health quotient (MHQ): development and usability study. JMIR Ment Health 7:e17935. 10.2196/17935 32706730 PMC7400040

[B21] Nolan H, Whelan R, Reilly RB (2010) FASTER: fully automated statistical thresholding for EEG artifact rejection. J Neurosci Methods 192:152–162. 10.1016/j.jneumeth.2010.07.015 20654646

[B22] Parameshwaran D, Subramaniyam NP, Thiagarajan TC (2019) Waveform complexity: a new metric for EEG analysis. J Neurosci Methods 325:108313. 10.1016/j.jneumeth.2019.108313 31278972

[B23] Parameshwaran D, Sathishkumar S, Thiagarajan TC (2021) The impact of socioeconomic and stimulus inequality on human brain physiology. Sci Rep 11:7439. 10.1038/s41598-021-85236-z 33811239 PMC8018967

[B24] Parameshwaran D, Bhavnani S, Mukherjee D, Sharma KK, Newson JJ, Subramaniyam NP, Divan G, Patel V, Thiagarajan TC (2025) Resting state EEG predicts developmental status in three year old children. Dev Cogn Neurosci 74:101575. 10.1016/j.dcn.2025.101575 40479750 PMC12173131

[B25] Parameshwaran D, Thiagarajan TC (2023) High variability periods in the EEG distinguish cognitive brain states. Brain Sci 13:1528. 10.3390/brainsci13111528 38002488 PMC10669877

[B26] Raven J (2000) The Raven’s progressive matrices: change and stability over culture and time. Cogn Psychol 41:1–48. 10.1006/cogp.1999.0735 10945921

[B27] Roser M, Ritchie H, Mathieu E (2024) Technological change. Our World in Data. Available at: https://ourworldindata.org/technological-change. Accessed September 16, 2024.

[B28] Sandre A, Troller-Renfree SV, Giebler MA, Meyer JS, Noble KG (2024) Prenatal family income, but not parental education, is associated with resting brain activity in 1-month-old infants. Sci Rep 14:13638. 10.1038/s41598-024-64498-3 38871945 PMC11176315

[B29] Singh A, Cole RC, Espinoza AI, Wessel JR, Cavanagh JF, Narayanan NS (2022) Evoked midfrontal activity predicts cognitive dysfunction in Parkinson’s disease. 2022.07.26.22278079. Available at: https://www.medrxiv.org/content/10.1101/2022.07.26.22278079v1. Accessed September 17, 2024.

[B30] Subramaniyam N, Thiagarajan T (2025) A novel method for estimating functional connectivity from EEG coherence potentials. Sci Rep 15:10723. 10.1038/s41598-025-94076-0 40155425 PMC11953265

[B31] Thompson PM, et al. (2020) ENIGMA and global neuroscience: a decade of large-scale studies of the brain in health and disease across more than 40 countries. Transl Psychiatry 10:1–28. 10.1038/s41398-020-0705-1 32198361 PMC7083923

[B32] Tomescu MI, Rihs TA, Rochas V, Hardmeier M, Britz J, Allali G, Fuhr P, Eliez S, Michel CM (2018) From swing to cane: sex differences of EEG resting-state temporal patterns during maturation and aging. Dev Cogn Neurosci 31:58–66. 10.1016/j.dcn.2018.04.011 29742488 PMC6969216

[B33] Valdes-Sosa PA, et al. (2021) The Cuban human brain mapping project, a young and middle age population-based EEG, MRI, and cognition dataset. Sci Data 8:45. 10.1038/s41597-021-00829-7 33547313 PMC7865011

[B34] Van Essen DC, Smith SM, Barch DM, Behrens TEJ, Yacoub E, Ugurbil K, WU-Minn HCP Consortium (2013) The WU-Minn Human Connectome Project: an overview. Neuroimage 80:62–79. 10.1016/j.neuroimage.2013.05.041 23684880 PMC3724347

[B35] Wang Y, Duan W, Dong D, Ding L, Lei X (2022) A test-retest resting, and cognitive state EEG dataset during multiple subject-driven states. Sci Data 9:566. 10.1038/s41597-022-01607-9 36100589 PMC9470564

[B36] Wilkinson CL, Yankowitz LD, Chao JY, Gutiérrez R, Rhoades JL, Shinnar S, Purdon PL, Nelson CA (2024) Developmental trajectories of EEG aperiodic and periodic components in children 2–44 months of age. Nat Commun 15:5788. 10.1038/s41467-024-50204-4 38987558 PMC11237135

[B37] Xiang C, Fan X, Bai D, Lv K, Lei X (2024) A resting-state EEG dataset for sleep deprivation. Sci Data 11:427. 10.1038/s41597-024-03268-2 38658675 PMC11043390

