# Supplementary figures and images for: EEG Data Quality in Large-Scale Field Studies in India and Tanzania
Source: eNeuro. 2025 Jul 23;12(7):ENEURO.0006-25.2025. doi: 10.1523/ENEURO.0006-25.2025 (PMC12309446; doi:10.1523/ENEURO.0006-25.2025)

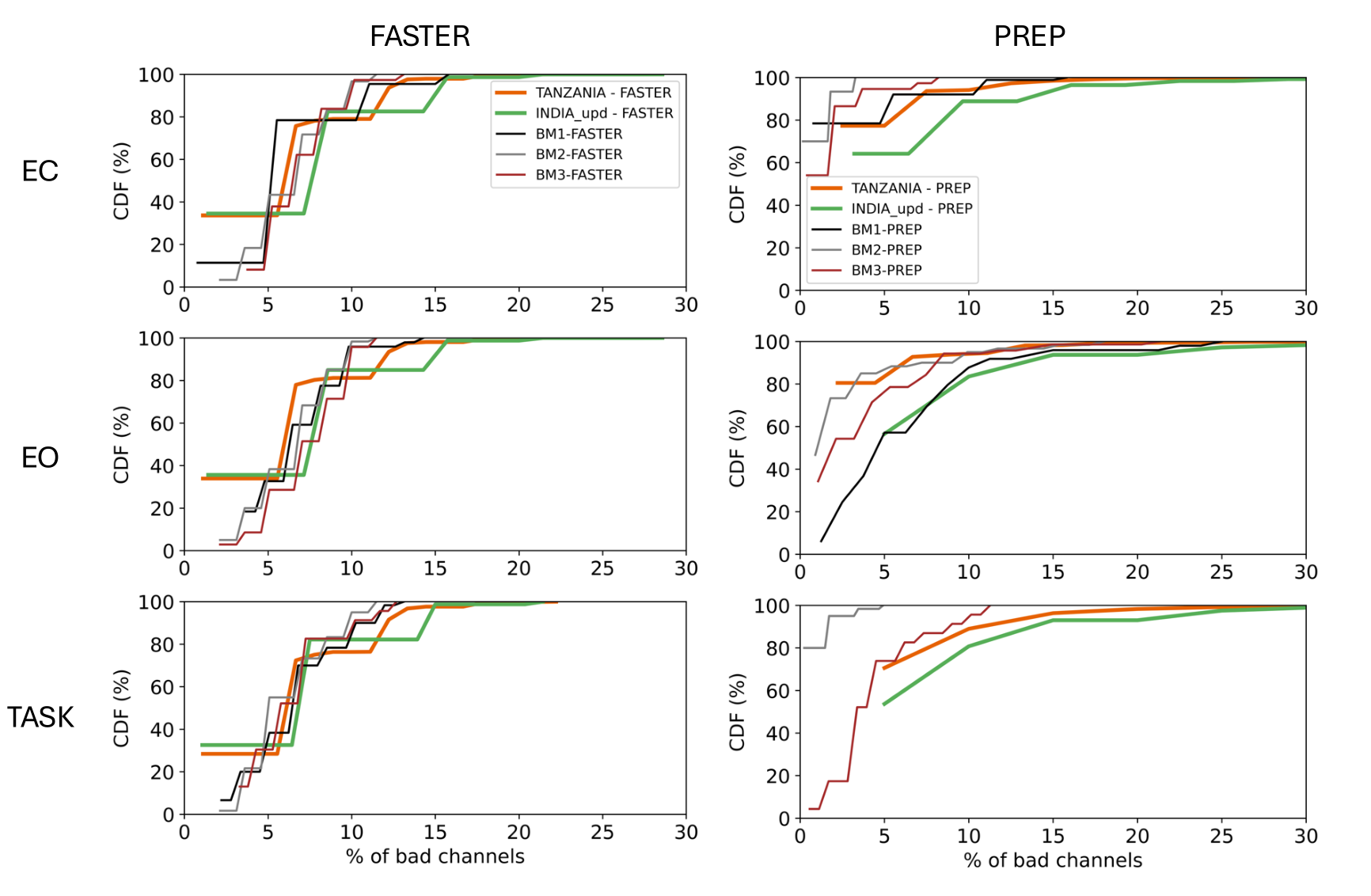

Supplement: Figure 3-1 — Cumulative distribution for the percentage of bad channels for FASTER (left) and PREP (right) for India, Tanzania and benchmark (BM) EEG datasets. Each row represents the EEG condition which include Eyes closed (EC, top), Eyes open (EO, middle) and TASK (bottom). Download Figure 3-1, TIF file. [file eneuro-12-ENEURO.0006-25.2025-s001.tif]

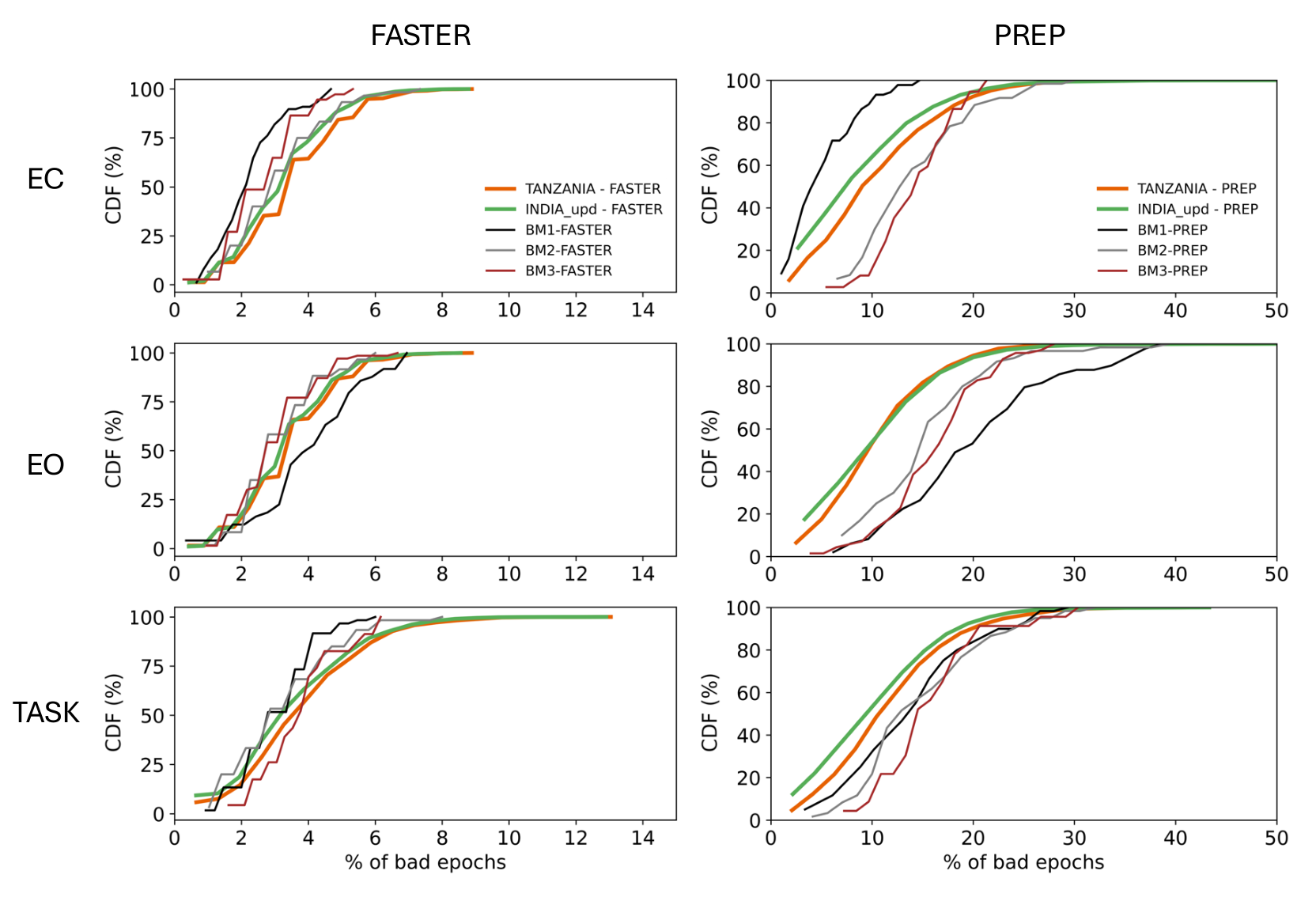

Supplement: Figure 4-1 — Cumulative distribution for the percentage of bad epochs for FASTER (left) and PREP (right) for India, Tanzania and benchmark (BM) EEG datasets. Each row represents the EEG condition which include Eyes closed (EC, top), Eyes open (EO, middle) and TASK (bottom). Download Figure 4-1, TIF file. [file eneuro-12-ENEURO.0006-25.2025-s002.tif]
